# Seroprevalence of Hepatitis B and C among Oncology Patients in Turkey

**DOI:** 10.3329/jhpn.v29i6.9903

**Published:** 2011-12

**Authors:** Sukran Kose, Ali Olmezoglu, Ayhan Gozaydin, Gulfem Ece

**Affiliations:** ^1^Department of Infectious Diseases and Clinical Microbiology, Tepecik Education and Research Hospital, Izmir, Turkey; ^2^Radiation Oncology Department, Ataturk Education and Research Hospital, Izmir, Turkey; ^3^Department of Infectious Diseases and Clinical Microbiology, Sanliurfa Education and Research Hospital, Sanliurfa, Turkey

**Keywords:** Cancers, Hepatitis, Hepatitis B, Hepatitis B virus, Hepatitis C, Hepatitis C virus, Seroprevalence, Turkey

## Abstract

Hepatitis B virus (HBV) is one of the public-health issues worldwide. Approximately two billion people are infected with HBV, and about 350 million people are chronic carriers globally. About 3% of the world population is infected with hepatitis C virus (HCV). Oncology patients receiving packed red blood cell suspensions and other blood products usually are in the high-risk group for infections due to these viruses. The aim of the study was to detect the seroprevalence of hepatitis B and hepatitis C among chemotherapy patients at the Oncology Department of the Tepecik Education and Research Hospital. HBsAg, anti-HBs, anti-HBcIgM, anti-HBc total and anti-HCV assays were studied by enzyme immunoassay method (Diasorin, Italy) in serum samples of patients (n=448) referred to the Department of Oncology of the Tepecik Education and Research Hospital during 1 June 2006–1 January 2007. Of the 448 patients, 19 (4.2%) were HBsAg-positive, and three (0.7%) had anti-HCV positivity. In this study, the seroprevalence of HBV was similar to previous data in Turkey. This could be due to widespread vaccination programmes. The seroprevalence of low anti-HCV may be because of controlled blood transfusion. Oncology patients should be monitored for their protective antibody levels against HBV, and they must be included in the vaccination programme. Their anti-HCV status should also be checked as well.

## INTRODUCTION

Hepatitis B virus (HBV), a worldwide health problem, can cause serious infections and has a carriage rate of 20% (1). Globally, four hundred million people are infected with HBV (http://www.who.int/mediacentre/factsheets/fs204/en/index.html). Almost 50% of them have cirrhosis or hepatocellular carcinoma. Approximately 3-5 million are carriers in Turkey (2). The seropositivity of HBsAg is 3.4% in the Western region while it is 8% in Eastern and Southeast Anatolia (3). Exposure to HBV increases with age in both urban and rural areas. Gurol *et al*. reported that the overall seroprevalence of HBV in Turkey was 4.19% (4). It is estimated that 3% of the world population is chronically infected with hepatitis C virus (HCV) (5). In Western Europe, the prevalence of HCV ranges from 0.4% to 3%. It is higher in Eastern Europe and the Middle East (6). The seroprevalence of HCV (1.2-4%) in Turkey is less than that of HBV, and this prevalence is less than 1% among blood donors (7).

Chemotherapy, which suppresses the immune system, can cause an increment in HBV DNA (viral load) and can damage the liver as the immune system cannot keep it under control (http://www.hbvadvocate.org/news/NewsUpdates_pdf/News_Review_2011/HBJ-8.4.pdf). Besides, chemotherapeutic medication can be directly hepatotoxic, and some chemotherapeutic agents and corticosteroids may damage the liver or may even lead to fulminant hepatitis and liver insufficiency by stimulating viral replication. In HBV genome, there is a specific area responding to corticosteroids and directly stimulating replication (8,9). It has been reported that high viral load before chemotherapy, HBeAg positivity, steroid-use, young age, male gender, lymphoma, or breast cancer can be risk factors for reactivation of HBV infection (10,11).

The level of hepatitis C viral RNA in blood has been shown to increase during chemotherapy and immunosuppression. At the same time, for those with pre-existing liver dysfunction, the transaminase levels often normalize during immunosuppression. After chemotherapy or immunosuppressant treatment, the hepatitis C viral RNA decreases with a simultaneous rise in transaminase levels (12).

The aim of our study was to evaluate the seroprevalence of hepatitis B and hepatitis C among patients with malignancy due to immunsuppression and also to evaluate the correlation of incidence of hepatitis and chemotherapy application/blood transfusion and association between the types of cancers and HBsAg/anti-HCV at the Oncology Department of the Tepecik Education and Research Hospital.

## MATERIALS AND METHODS

Patients admitted to the oncology outclinic of the Tepecik Education and Research Hospital, during 1 June 2006—1 January 2007, were included in the study. This study included 448 outclinic patients. Enlighted approval was taken from these patients. Serum samples were obtained to study HBsAg, anti-HBs, anti-HBc IgM, anti-HBc total, and anti-HCV assays by enzyme immunoassay method (EIA) (Etimax, Diasorin, Italy) at the Infectious Diseases and Clinical Microbiology Laboratory of the Tepecik Education and Research Hospital. Statistical analysis of correlation between chemotherapy application/blood transfusion and the incidence of hepatitis and also between types of cancer and HBsAg/anti-HCV seroprevalence was performed using the SPSS software (version 13.0).

## RESULTS

Of the 448 patients, 317 (70.8%) were women and 131 (29.2%) were men. Their mean age was 54 years. Types of cancer detected included breast cancer (57%), gastrointestinal system cancers (24.2%), and head-neck cancers (6.4%). Other types of cancers included lung, gynaecological and genitourinary system, soft tissue, and skin cancers, and cancer with unknown primary localization. The mean duration of diagnosis of cases was 29.7 months. Chemotherapy was not administered in 5.7% of the cases. Blood transfusion took place in 36.5% of the cases. The positivity of HBsAg was determined in 19 (4.2%) individuals, and the positivity of anti-HCV was found in three (0.7%) patients. HBsAg was negative in 429 (95.8%) patients. Anti-HBc total was positive in 172 (38.4%) patients. Thirty-one (18.1%) patients had isolated anti-HBc total positivity, and 155 patients (34,6%) had anti-HBs positivity (Table).

**Table.** Presentation of hepatitis B and C serological parameters of oncology patients (n=448)

Hepatitis marker	Positive		Negative	
	No.	%	No.	%
HBsAg	19	4.2	429	95.8
Anti-HBs	155	34.6	293	65.4
Anti-HBc total	172	38.4	276	61.6
Anti-HCV	3	0.7	445	99.3

No significant correlation was found between chemotherapy application/blood transfusion and the incidence of hepatitis. No significant association was determined between types of cancers and HBsAg/anti-HCV.

## DISCUSSION

In patients receiving chemotherapy, serious problems, such as delay in treatment, decrease in dose concentration, and even treatment interruption or fulminant hepatitis have been observed due to reactivation of hepatitis (8,9,13).

In our study, HBsAg positivity was determined as 4.2% and anti-HCV positivity as 0.7%. Anti-HBc total was found to be positive in 172 patients while 31 of these patients had isolated anti-HBc total positivity. In our study, 155 (34,6%) patients had anti-HBs positivity.

Kebudi *et al.* compared the prevalence of pre-and post-treatment hepatitis B and C in 50 paediatric oncology patients who received multiple transfusions and intensive chemotherapy, and they determined that children infected with HBV during immunosuppressive therapy are at a greater risk of becoming chronic carriers, and precautions must be taken for immunization of these children after treatment (14). Monteleone *et al*. reported the seroprevalence (8.9%) of hepatitis C among paediatric oncology patients who received blood transfusions (15). Demirkaya *et al.* investigated the trend in frequency after the routine application of hepatitis B vaccine and to determine the frequency of hepatitis B and C infections (16). They found HBsAg seropositivity in one of 94 lymphoma and solid tumour patients; none had developed hepatitis C but one of them got HCV during follow-up (16).

Kocabas *et al*. reported that a higher seroprevalence of HBV and HCV among paediatric patients with cancer (17). Topeli *et al*. reported HBsAg positivity in 11 (13.2%) of 83 cancer patients, anti-HCV positivity in 10 (12.0%) patients, and both HBsAg and anti-HCV positivity were detected in one (1.2%) patient (18). Utkan *et al*. reported that oncology patients had HBsAg and anti-HCV positivity (4.8% and 2.8% respectively) (19).

Malaguernera *et al.* reported the seroprevalence of anti-HCV among 236 elderly cancer patients compared to 300 elderly volunteers. Of the 236 elderly cancer patients, 87 (36%) were positive for HCV antibodies, and 32 (10%) of the 300 elderly patients were positive for hepatitis C. A comparison between the two groups showed the significant difference (p<0.001) between patients with kidney cancer, bladder cancer, or prostate cancer and the control group. These patients were more prone to acquire HCV infection because of their frequent hospitalizations, and the immunological changes in patients with tumours may lower their threshold for HCV infection (20).

Uzun *et al*. reported the prevalence (6.7%) of anti-HCV positivity among lung cancer patients, which was significantly higher compared to the normal Turkish population (21).

Eren *et al*. reviewed the medical records of 4,400 patients undergoing cytotoxic chemotherapy at the Medical Oncology Department of the Selcuk University for the July 2004—July 2007 period (22). In total, 1,826 patients had records of hepatitis B serology. They found HBsAg seropositivity (about 5%), which is very close to the estimated seroprevalence of hepatitis B in Turkey. Hepatitis B reactivated in 15% of patients who were undergoing cytotoxic chemotherapy (22).

In our study, the seroprevalence of HBV was similar to previous data in Turkey. Anti-HCV positivity was found in three (0.7%) patients. The low seroprevalence of HBV could be due to ongoing vaccination programmes and screening of blood products for HCV and HBV. Also, the widespread vaccination programmes against HBV can be another reason for the low seroprevalence of HBV. There was no significant correlation between chemotherapy application/blood transfusion and the incidence of hepatitis. No significant association was observed between types of cancers and HBsAg/anti-HCV. HBsAg assay should be done in all patients before chemotherapy or immunosuppressive treatment. These patients undergo invasive procedures and blood product transfusions, and because of this, hepatitis seropositivity develops. This situation can be followed by a risk of reactivation and fulminant hepatitis after chemotherapy. Seronegative patients need to receive vaccination against HBV (23). Screening for HBV is required before chemotherapy, and prophylactic antiviral therapy can reduce not only the incidence of HBV reactivation but also HBV-related morbidity and mortality. On the other hand, the introduction of more sensitive screening tests and stringent donor-selection procedures has decreased the incidence of HCV infection but there is still a risk for HCV infection; so, these immunosuppressed patients should also be monitored for HCV.
